# Tripartite motif 10 regulates cardiac hypertrophy by targeting the PTEN/AKT pathway

**DOI:** 10.1111/jcmm.15257

**Published:** 2020-04-28

**Authors:** Hui Yang, Xiao‐Xiao Wang, Chun‐Yu Zhou, Xue Xiao, Cui Tian, Hui‐Hua Li, Chun‐Lin Yin, Hong‐Xia Wang

**Affiliations:** ^1^ Department of Physiology and Pathophysiology School of Basic Medical Sciences Capital Medical University Beijing China; ^2^ Beijing Key Laboratory of Metabolic Disorders Related Cardiovascular Diseases Capital Medical University Beijing China; ^3^ Department of Cardiology Institute of Cardiovascular Diseases First Affiliated hospital of Dalian Medical University Dalian China; ^4^ Department of Cardiology Xuan Wu Hospital Capital Medical University Beijing China

**Keywords:** AKT, Cardiac hypertrophy, PTEN, Transverse aortic constriction, TRIM10

## Abstract

The pathogenesis of cardiac hypertrophy is tightly associated with activation of intracellular hypertrophic signalling pathways, which leads to the synthesis of various proteins. Tripartite motif 10 (TRIM10) is an E3 ligase with important functions in protein quality control. However, its role in cardiac hypertrophy was unclear. In this study, neonatal rat cardiomyocytes (NRCMs) and TRIM10‐knockout mice were subjected to phenylephrine (PE) stimulation or transverse aortic constriction (TAC) to induce cardiac hypertrophy in vitro and in vivo, respectively. Trim10 expression was significantly increased in hypertrophied murine hearts and PE‐stimulated NRCMs. Knockdown of TRIM10 in NRCMs alleviated PE‐induced changes in the size of cardiomyocytes and hypertrophy gene expression, whereas TRIM10 overexpression aggravated these changes. These results were further verified in TRIM10‐knockout mice. Mechanistically, we found that TRIM10 knockout or knockdown decreased AKT phosphorylation. Furthermore, we found that TRIM10 knockout or knockdown increased ubiquitination of phosphatase and tensin homolog (PTEN), which negatively regulated AKT activation. The results of this study reveal the involvement of TRIM10 in pathological cardiac hypertrophy, which may occur by prompting of PTEN ubiquitination and subsequent activation of AKT signalling. Therefore, TRIM10 may be a promising target for treatment of cardiac hypertrophy.

## 1 INTRODUCTION

Sustained pathological cardiac hypertrophy, which is characterized by cell size enlargement and cardiac contractility dysfunction with subsequent interstitial fibrosis, eventually progresses to heart failure.^1^, ^2^ Extracellular stress, including biomechanical stress and neurohumoral mediators, activates intracellular hypertrophic signalling pathways, thus leading to an imbalance of proteins synthesis and degradation.^3^, ^4^ Although many studies have demonstrated the involvement of protein ubiquitination pathways in cardiac hypertrophy,^5^ the molecular mechanism has not yet been fully clarified.

TRIM10, a member of both the tripartite motif (TRIM) family protein and E3 ubiquitin ligase complex, contains an N‐terminal RING finger/B‐box/coiled coil motif.^6^ TRIM family proteins are implicated in numerous biological processes including apoptosis, growth, differentiation, development, cell cycle regulation and senescence.^7^ TRIM family proteins are involved in many human diseases including immunological, neurological, musculoskeletal and cardiovascular diseases, as well as cancer.^6^ The most‐studied TRIM family member in the heart is muscle RING fingers (TRIM63/55/54, also known as MuRF1/2/3).^8^ In addition, mounting evidence has demonstrated that many TRIMs, including TRIM8/21/24/32/45/67/72, play important roles in cardiac function and disease, for example by regulating cardiomyocyte differentiation and apoptosis, cardiac atrophy, ischemia‐reperfusion injury, and dilated and hypertrophic cardiomyopathies.^8^, ^9^, ^10^ TRIM10, another member of the TRIM family, is essential for the differentiation and survival of terminal erythroid cells.^11^ However, functional roles and molecular mechanisms of TRIM10 during cardiac hypertrophy remain to be elucidated.

In this study, we aimed to examine whether TRIM10 is involved in cardiac hypertrophy and, if so, its molecular target. We showed that TRIM10 was up‐regulated in both mouse heart tissue and neonatal rat cardiomyocytes (NRCMs) subjected to hypertrophic stresses. TRIM10 knockdown significantly prevented phenylephrine (PE)‐stimulated enlargement of cells, whereas TRIM10 overexpression aggravated this effect in vitro. Furthermore, this effect was confirmed following transverse aortic constriction (TAC)‐induced cardiac remodelling in mice. In addition, we found that the effect regulated by TRIM10 was mediated by interactions with phosphatase and tensin homolog (PTEN); specifically, TRIM10 promoted PTEN ubiquitination, thus leading to AKT signalling activation. Taken together, our results identify TRIM10 as a potential novel regulator involved in pathological cardiac hypertrophy, mainly through its action on PI3K/AKT signalling.

## 2 MATERIALS AND METHODS

### 2.1 Generation TRIM10‐knockout mice and pressure overload model

Conventional Trim10‐knockout mice were generated by Cyagen Biosciences (Guangzhou, China). In brief, the mouse *Trim10* (*mTrim10*) gene contains seven exons; exon 1 of *mTrim10* was chosen for targeting using a TALEN‐mediated genome‐editing approach. Homozygous Trim10‐knockout mice were generated through breeding of heterozygous mice to each other. All mice were housed in a specific pathogen‐free condition animal facility at Capital Medical University (Beijing, China).

The pressure overload model was established by TAC surgical operation as previously described.^12^, ^13^ Briefly, male wild‐type C57BL/6J mice and TRIM10‐knockout mice aged 10‐12 weeks were randomized into TAC and Sham groups. For anaesthesia, mice were intraperitoneally administered 0.25 mg/g tribromoethanol. After cutting off the manubrium sternum, the exposed transverse aortic arch was then ligated between the innominate artery and left common carotid artery using a 6‐0 silk suture. A 27‐gauge blunt needle was tied against the aorta, and the skin was closed after removal of this needle. Mice were housed under standard conditions with 12‐hour light/dark cycles. All experiments were performed according to the guidelines of and approved by the Animal Subjects Committee of Capital Medical University.

### 2.2 Transthoracic echocardiography

In vivo cardiac geometry and function was assessed using a Vevo 770 high‐resolution micro‐imaging system (VisualSonics), as previously described.^14^ Two weeks after TAC surgery, two‐dimensional and M‐mode imaging were performed. The left ventricle inner diameter during diastole (LVIDd) and left ventricle anterior wall thickness during diastole (LVAWd) of mouse hearts were measured. In addition, left ventricular ejection fraction (EF) and fractional shortening (FS) were evaluated.

### 2.3 Histopathology and immunofluorescence

Two weeks after TAC surgery, hearts were harvested from mice. Isolated hearts were photographed using a stereo microscope (SZ61; Olympus). Next, hearts were fixed in 4% paraformaldehyde, embedded in paraffin, cut into 5‐μm slices and mounted onto glass microscope slides. As previously described, slides were subjected to haematoxylin and eosin, Masson's trichrome and wheat germ agglutinin staining techniques.^15^ Each heart sample was imaged at 100× or 200× magnification of 15‐20 random fields. The surface area of cells was calculated by measuring 150‐200 cells per slide. Fibrotic areas of heart slices were analysed by Image J Software (imagej.nih.gov).

### 2.4 Cell culture

Primary rat cardiomyocytes were isolated from 1‐day‐old Sprague Dawley neonatal rat hearts, as previously described.^16^ Briefly, isolated hearts were minced and treated with 0.25% trypsin in a 37ºC water bath. The resulting cells were resuspended in Dulbecco's Modified Eagle Medium (DMEM; Gibco) containing 10% foetal calf serum (FCS, Gibco). Cardiomyocytes were selectively separated from neonatal cardiac fibroblasts after allowing the latter to adhere to plates for 30 minutes. Cardiomyocytes were cultured in DMEM containing 10% FCS and 1% streptomycin/penicillin (Gibco) at 37°C in a 5% CO_2_ incubator. Cells were pre‐treated with siRNA‐TRIM10, Ad‐Trim10, scramble‐siRNA or Ad‐GFP (Hanbio, Shanghai, China) for 48 hours, followed by stimulation with PE (30 μmol/L, Sigma‐Aldrich) or phosphate‐buffered saline (PBS) for another 24 hours. Hypertrophy was analysed by α‐actinin (CST, MA, USA) immunofluorescence staining.

### 2.5 Quantitative RT‐PCR (qRT‐PCR) analyses

Total RNA was extracted with TRIzol reagent (Invitrogen) as previously described.^17^ Total RNA (1 μg) was reverse‐transcribed to generate cDNA with a GoScript™ Reverse Transcription System (Promega). SYBR Green PCR Master Mix (Applied Biosystems) was used to detect and perform relative quantification of the indicated genes. Expression data were normalized to GAPDH. Primers for atrial natriuretic factor (ANF), B‐type natriuretic peptide (BNP), myosin heavy chain beta (β‐MHC), collagen I and collagen III were designed as previously described.^15^ Primer sequences are listed in Table S1.

### 2.6 Western blotting

Frozen heart tissue or cultured cells were lysed in radioimmunoprecipitation assay (RIPA) buffer (PMSF:RIPA = 1:100; Solarbio Science Technology). The resulting suspensions were subjected to sodium dodecyl sulphate‐polyacrylamide gel electrophoresis, followed by transfer to polyvinylidene difluoride membranes by immunoblotting (Bio‐Rad). Chemiluminescence was visualized with an ECL kit (Millipore). Western blot bands were measured with ImageJ software using GAPDH (1:5000) as an internal loading control. All primary antibodies (Trim10, PTEN, phospho‐AKT, AKT, phospho‐ERK1/2, ERK1/2, phospho‐p38 MAPK, 38 MAPK, phospho‐JNK, JNK, phospho‐STAT3, STAT3, calcineurin and SHP1) were incubated at a dilution of 1:800‐1000, while secondary antibodies were diluted at 1:2000 or 1:5000; all antibodies were purchased from Cell Signaling Technology.

### 2.7 Co‐immunoprecipitation assays (Co‐IP)

To determine whether TRIM10 interacts with PTEN, co‐IP was performed as previously described.^16^ The primary antibody was immobilized on protein A/G magnetic beads (Bimake) according to the manufacturer's instructions. Next, cell lysates of cardiac tissue were added to the protein A/G magnetic beads and rotated at 4°C overnight. The beads were washed with 10‐50 μL of wash buffer (0.1‐0.2 mol/L glycine, 0.1%‐0.5% NP‐40, pH 7.5), and the resulting supernatant was collected. Whole tissue lysates and protein precipitates were evaluated by Western blot. Membranes were immunoblotted with the following antibodies: mouse anti‐PTEN (Cat. No. 9556, Cell Signaling Technology), mouse anti‐k48‐linkage specific polyubiquitin (Cat. No. 12805, Cell Signaling Technology) and rabbit anti‐TRIM10 (Cat. No. ab151306, Abcam).

### 2.8 Statistical analysis

All results were expressed as mean ± SEM and analysed using Prism7 (GraphPad Software). Statistical analysis was performed using an unpaired, two‐tailed student's *t* test and one‐way ANOVA of variance. *P* < .05 was treated as statistically significant.

## 3 RESULTS

### 3.1 *TRIM10 knockdown attenuated PE‐induced cardiomyocyte hypertrophy *in vitro*, and its overexpression aggravated this effect*

**FIGURE 1:**
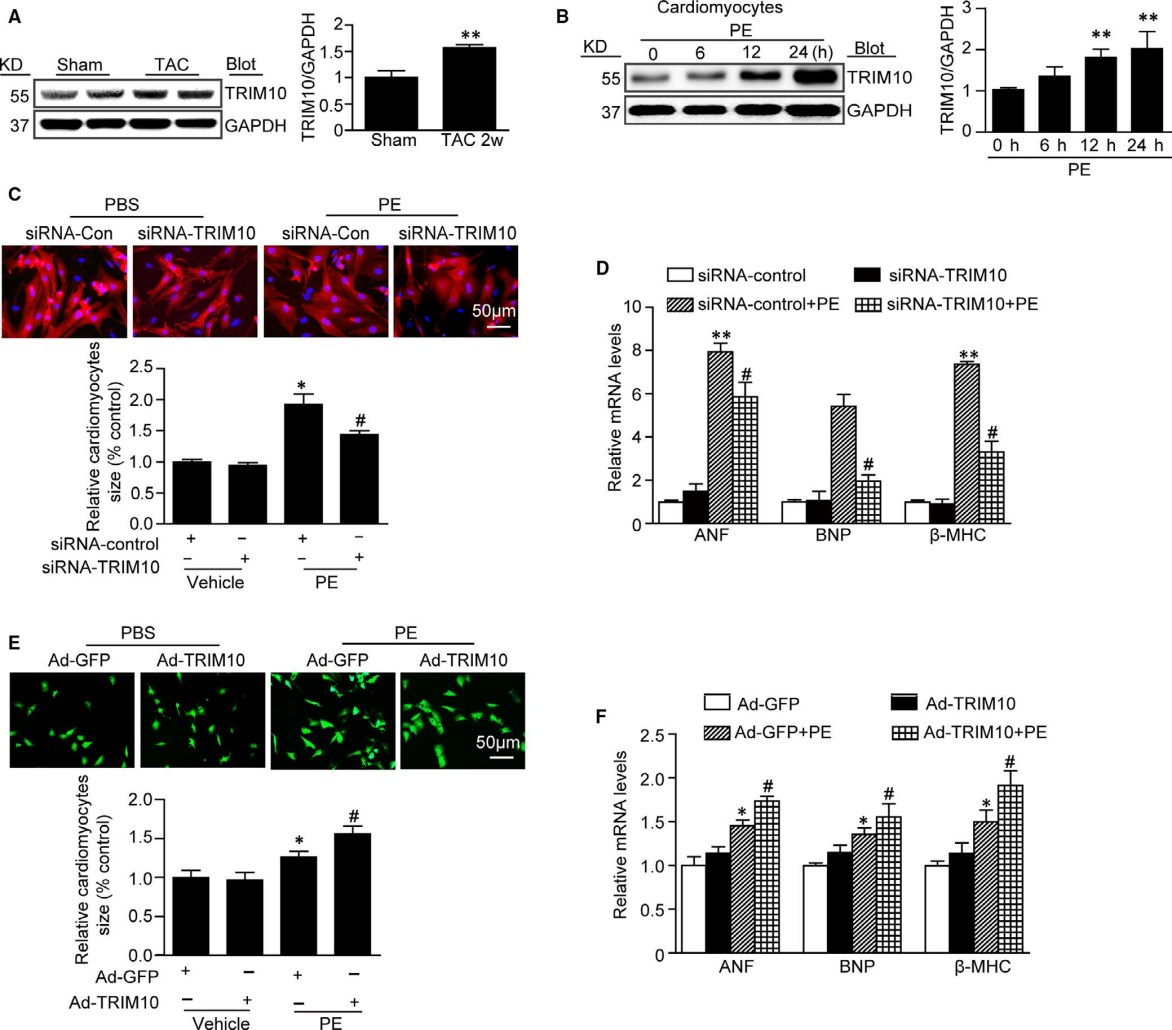
TRIM10 knockdown attenuates PE‐induced cardiomyocyte hypertrophy in vitro, and its overexpression aggravates this effect. A, Western blotting detected the expression of TRIM10 in C57BL/6 wild‐type (WT) mice hearts subjected to TAC surgery or Sham operation for 2w. B, Western blotting detected the expression of TRIM10 in PE‐treated neonatal rat cardiomyocytes (NRCMs) at different time‐points. C, NRCMs were transfected with siRNA‐TRIM10 or siRNA‐Control for 72 h and then treated with PE for additional 24 h. Double immunostaining (red for α‐actinin, blue for DAPI) was used to evaluate the NRCMs size. D, qRCR detected the mRNA expression of hypertrophic markers such as ANF, BNP and β‐MHC after siRNA‐TRIM10infection. E, NRCMs were transfected with ad‐TRIM10 or ad‐GFP for 72 h and then treated with PE for additional 24 h. Double immunostaining (red for α‐actinin, blue for DAPI) was used to evaluate the NRCMs size. F, qRCR detected the mRNA expression of hypertrophic markers such as ANF, BNP and β‐MHC after ad‐TRIM10 infection. Data are mean ± SEM (n = 3‐5 per group). *P < .05, **P < .01 vs Sham/PBS group, ^#^ *P* < .05 vs PE group

To elucidate the role of TRIM10 in cardiac hypertrophy, C57BL/6 wild‐type mice were subjected to TAC surgery to induce cardiac hypertrophy. Compared with Sham‐treated hearts, TRIM10 was significantly up‐regulated in the TAC group (Figure 1A). Moreover, changes in TRIM10 expression were further verified in NRCMs in a time‐dependent manner after PE treatment (Figure 1B). Altogether, these results suggest that TRIM10 expression is increased in cardiomyocytes responding to hypertrophic stimuli and may be involved in the pathological process of cardiac hypertrophy.

We next investigated whether TRIM10 could modulate the development of cardiac hypertrophy by transfecting NRCMs with siRNA‐TRIM10, Ad‐Trim10, scramble‐siRNA (as siRNA‐con) or Ad‐GFP. Cardiomyocytes were administered either PE (30 μmol/L) or PBS (as a control) for 24 hours. Compared with the scramble‐siRNA group, siRNA‐TRIM10 knockdown reduced cardiomyocyte surface area and mRNA expression of ANP, BNP and β‐MHC (Figure 1C,D). Conversely, Ad‐TRIM10 infection markedly aggravated the effects induced by PE stimulation (Figure 1E,F). These in vitro data suggest that TRIM10 may act as a pro‐hypertrophic factor.

### 3.2 TRIM10 deficiency alleviated TAC‐induced cardiac hypertrophy

**FIGURE 2:**
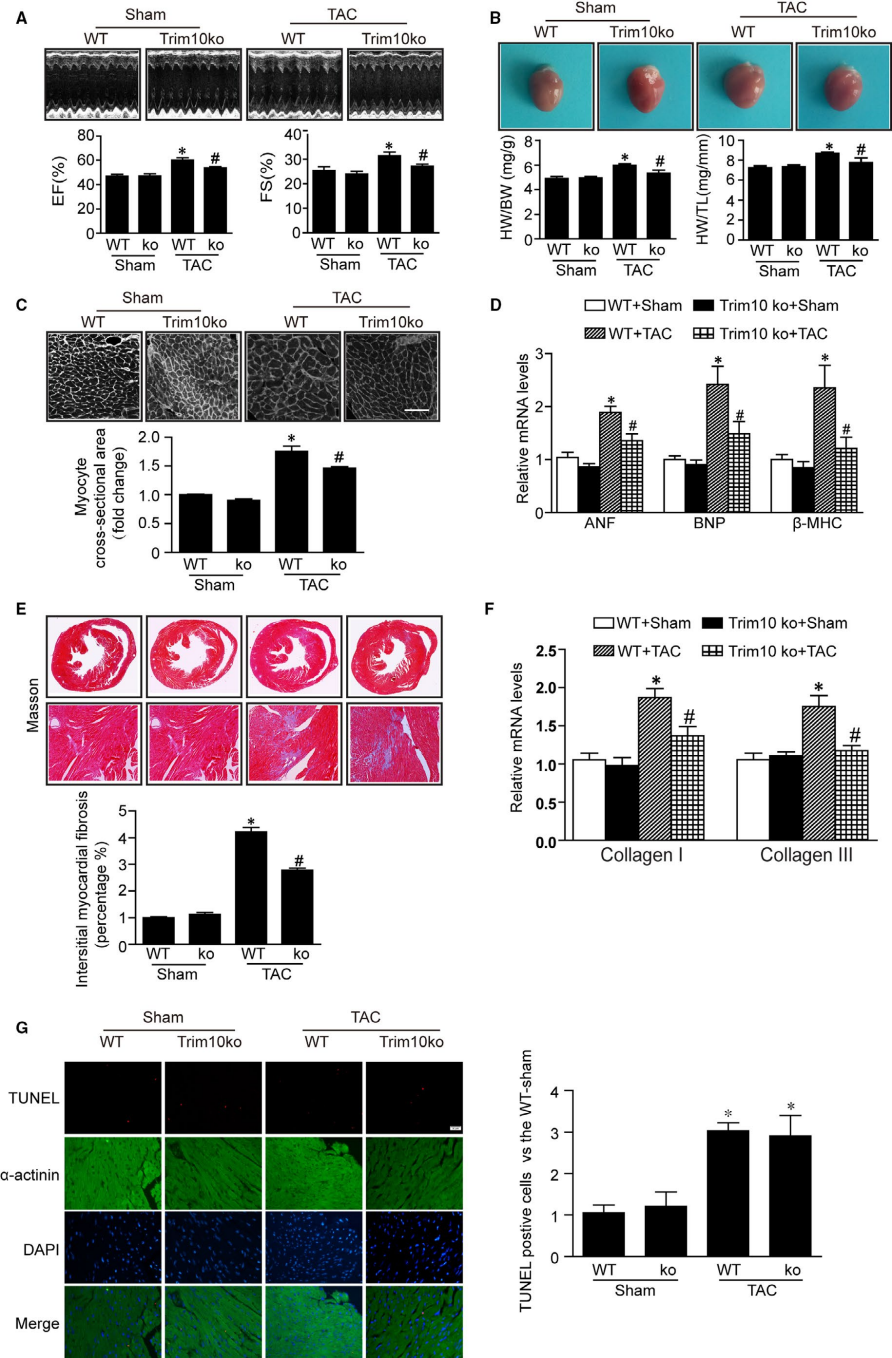
TRIM10 deficiency alleviates TAC‐induced cardiac hypertrophy. A, TRIM10ko and WT mice were subject to TAC or Sham surgery for 2w. Ejection fraction (EF%) and fractional shortening (FS%) were assessed by M‐mode echocardiograms. B, The representative heart photographs of each group were showed, and heart weight/bodyweight (HW/BW) and heart weight/tibia length (HW/TL) ratios were calculated. C, The cell size was examined by WGA staining. D, The mRNA levels of hypertrophic markers such as ANF, BNP and β‐MHC were detected by qPCR. E, The interstitial fibrosis areas were calculated by Masson's staining of heart sections (×10 and ×200). F, The mRNA levels of collagen I and III in the hearts were detected by qPCR. G, The apoptosis were green, myocardial tissue were identified by a‐actinin antibody staining (red), and nuclei by DAPI staining (blue) (×200).Data are mean ± SEM (n = 6 per group). *P < .05 vs Sham group, ^#^ *P* < .05 vs TAC group

To further verify the effect of TRIM10 on cardiac hypertrophy, TRIM10‐knockout mice were generated and subjected to TAC surgery. The results of echocardiography analysis 14 days after TAC showed cardiac pump function was increased, including EF and FS. However, this induction was ameliorated in the hearts of TRIM10‐knockout mice (Figure 2A). Concomitantly, TRIM10‐knockout mice also exhibited decreased TAC‐induced hypertrophic responses, such as heart weight/bodyweight (HW/BW) ratio, heart weight/tibia length (HW/TL) ratio, myocyte size and expression of hypertrophic markers ANF, BNP and β‐MHC (Figure 2B–D). Because cardiac fibrosis is secondary to hypertrophy, fibrotic areas and mRNA expression of collagen I and III were evaluated. The results demonstrated that TRIM10 knockout alleviated TAC‐induced increases in fibrotic area and collagen I/III mRNA expression (Figure 2E,F). In addition, we also observed whether TRIM10 knockout can affect the number of TUNEL‐positive cells in hearts, and the results showed TRIM10 knockout did not further increased apoptosis induced by TAC treatment. Altogether, these in vivo data further support the involvement of TRIM10 in a pro‐hypertrophic effect.

### 3.3 TRIM10 knockout or knockdown inhibited AKT activation and STAT3 signalling pathways

**FIGURE 3:**
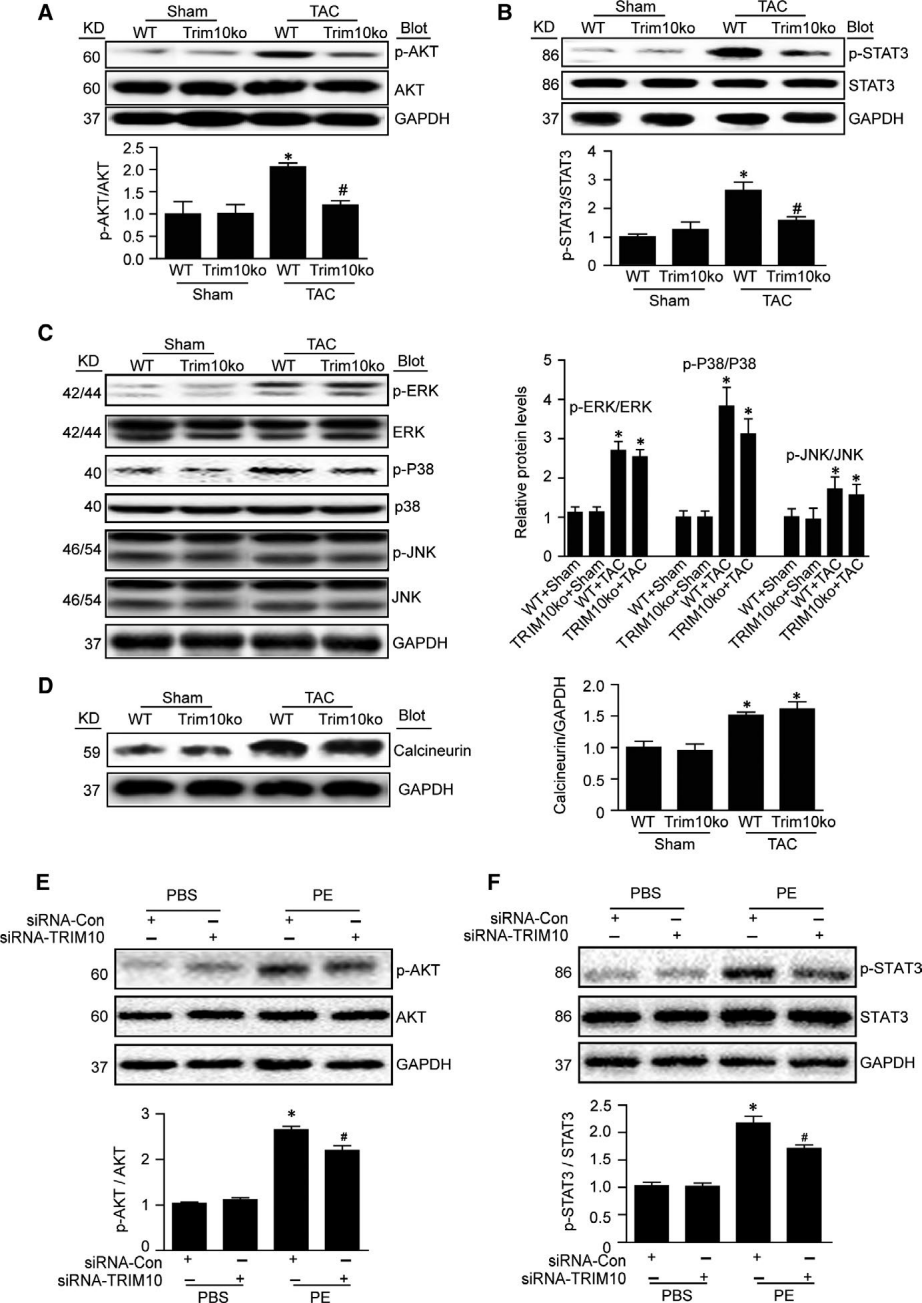
TRIM10 deficiency or knockdown inhibits the activation of AKT and STAT3 signalling pathways. A, The ratio of p‐AKT/AKT was analysed in the hearts of mice treated as in Figure 2A. B, The ratio of p‐STAT/STAT. C, The ratios of p‐ERK1/2/ERK1/2, p‐P38/P‐38 and p‐JNK/JNK. D, The expression of calcineurin. E, NRCMs were treated as in Figure 1C. The ratio of p‐AKT/AKT in NRCMs treated as in Figure 1C. F, The ratio of p‐STAT/STAT in NRCMs treated as in Figure 1C. GAPDH was used as a loading control. Data are mean ± SEM (n = 3‐4).*P < .05 vs Sham or PBS group; ^#^ *P* < .05 vs TAC group or PE group

To elucidate possible molecular mechanisms of TRIM10, we examined multiple classical signalling pathways potentially involved in cardiac hypertrophy under pressure load. Western blot assays revealed that 2‐week TAC treatment significantly induced phosphorylation of AKT, Stat3 and MAPKs (including ERK/P38/JNK), as well as calcineurin expression compared with Sham controls. In contrast, TRIM10 knockout dramatically reversed AKT and STAT3 phosphorylation levels, but had no effect on MAPKs or calcineurin (Figure 3A–D). Furthermore, changes in AKT and STAT3 phosphorylation were verified in NRCMs transfected with siRNA‐TRIM10 (Figure 3E,F). According to these results, we speculate that TRIM10‐mediated pathological cardiac hypertrophy may be associated with AKT‐ or STAT3‐dependent signalling.

### 3.4 TRIM10 knockout or knockdown decreased PTEN degradation, and blocking of PTEN activity up‐regulated AKT phosphorylation

**FIGURE 4:**
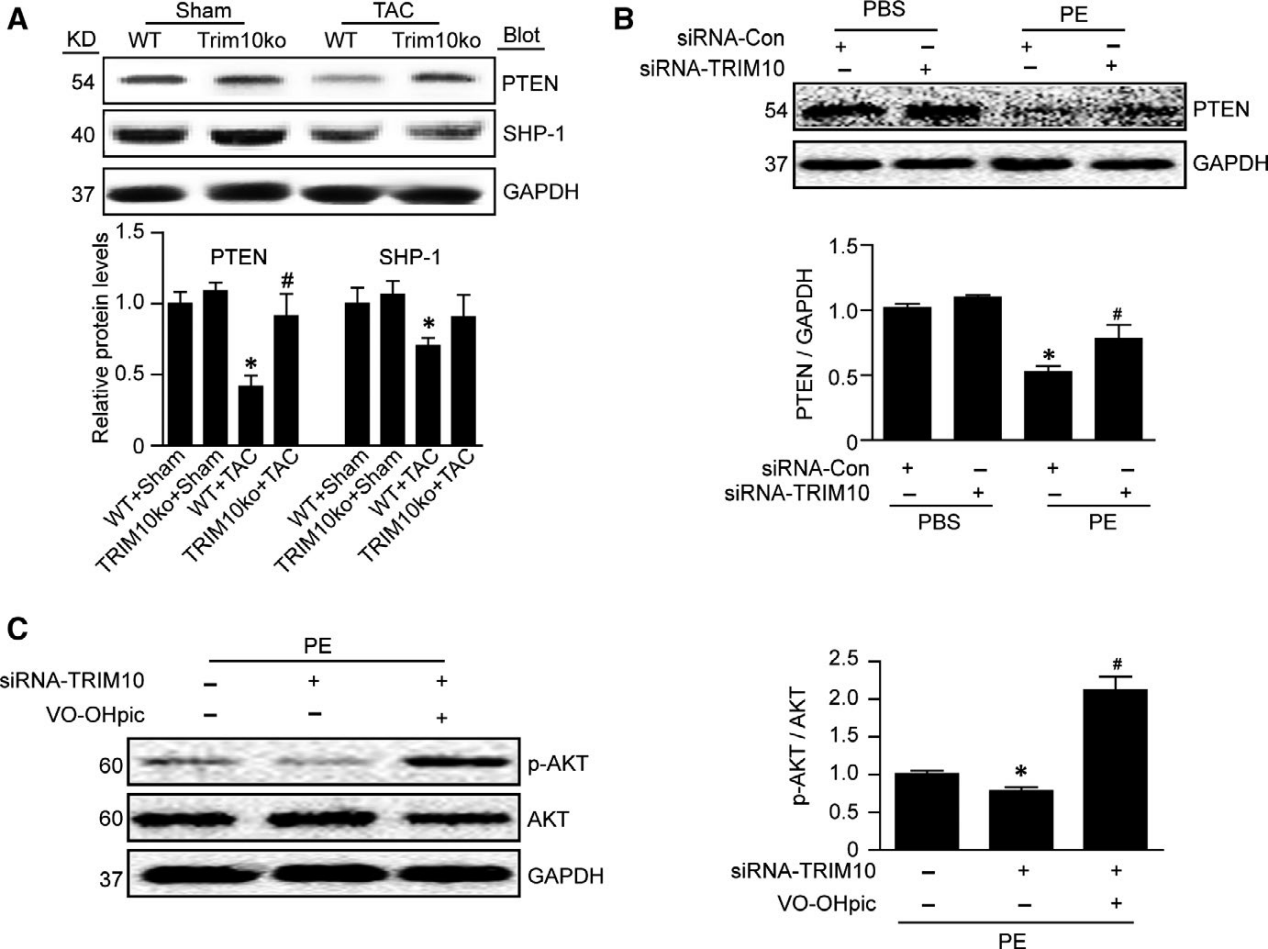
TRIM10 deficiency or knockdown reduced the expression of PTEN, and blocking of PTEN activity up‐regulates the phosphorylation of AKT. A, The expressions of PTEN and SHP‐1 were detected in the hearts of mice treated as in Figure 2A. *P < .05 vs Sham group; ^#^ *P* < .05 vs TAC group.B, The expression of PTEN was detected in the NRCMs treated as in Figure 1C. *P < .05 vs PBS group; ^#^ *P* < .05 vs PE group. C, NRCMs were transfected with siRNA‐TRIM10 and then stimulated by PE for 24 h with or without PTEN inhibitor VO‐OHpic (5 μmol/L). The ratio of p‐AKT/AKT was analysed. GAPDH was used as a loading control. Data are mean ± SEM (n = 3‐4).* *P* < .05 vs PE; ^#^ *P* < .05 vsPE+VO‐OHpic

PTEN is known to dephosphorylate PIP3 to generate PIP2, thus inhibiting AKT activation,^18^ and SHP1 can negatively regulate STAT3 activation.^19^ In addition, PTEN and SHP1 are modulated by the ubiquitin‐proteasome system. In this study, Western blot results showed that PTEN protein expression was down‐regulated after TAC surgery in mice or PE stimulation of NRCMs. However, this effect was markedly reversed in TRIM10‐knockout mice or TRIM10‐knockdown NRCMs, and TRIM10‐knockout or knockdown only slightly increased PTEN expression, whereas, TRIM10 knockout had no effect on TAC‐induced down‐regulation of SHP1 (Figure 4A,B). We further assessed whether PTEN directly mediated the activation of AKT by administering NRCMs the selective PTEN inhibitor VO‐OHpic. After PE stimulation for 24 hours, expression of phosphorylated AKT was markedly decreased in TRIM10‐knockdown cardiomyocytes compared with scramble‐siRNA, and this decrease was significantly reversed in VO‐OHpic‐treated cardiomyocytes transfected with siRNA‐TRIM10 (Figure 4C). These results indicate that TRIM10 induced AKT activation by degrading PTEN.

### 3.5 TRIM10 knockout decreased TAC‐induced PTEN ubiquitination

**FIGURE 5:**
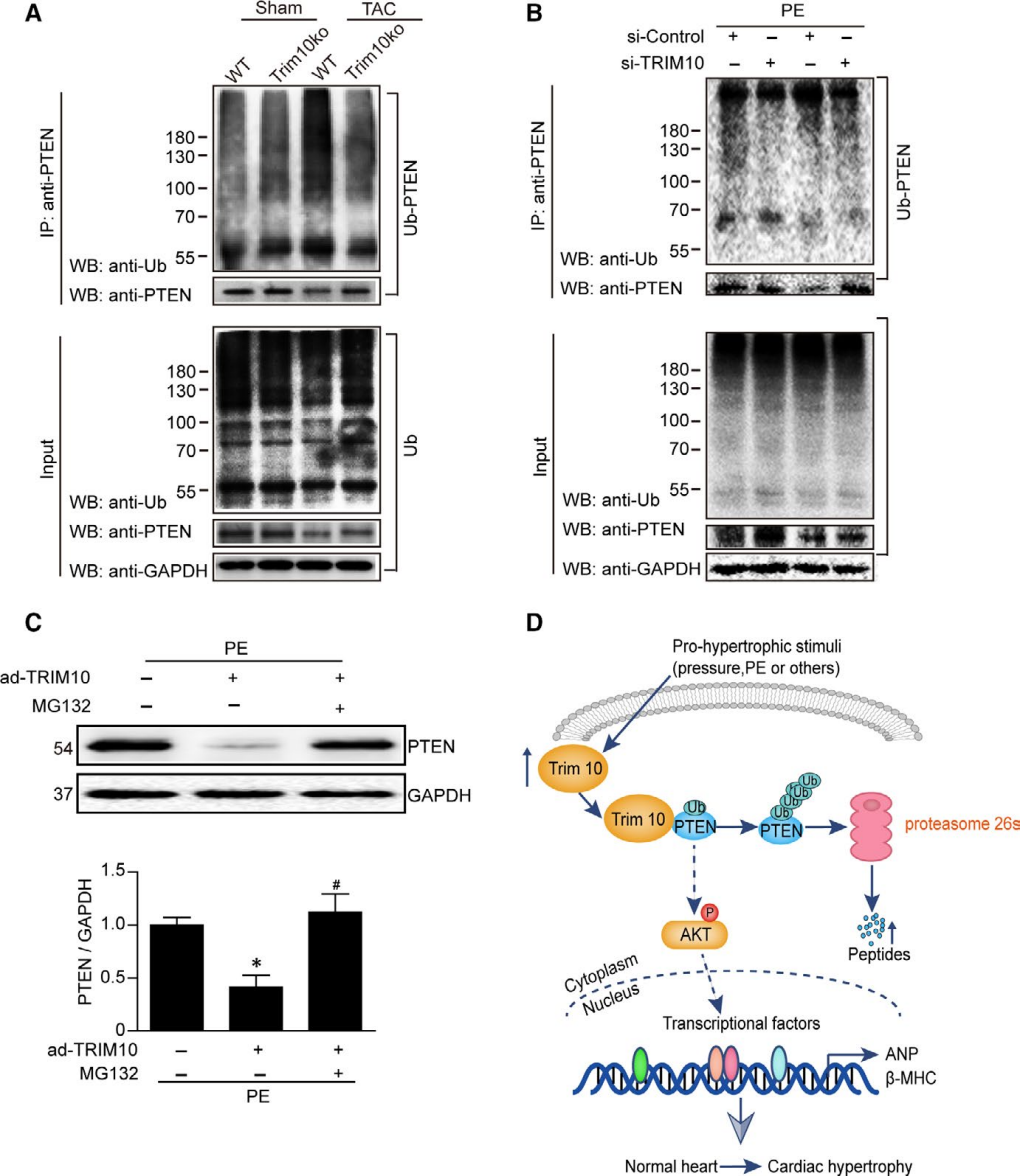
TRIM10 knockout decreased PTEN degradation and ubiquitination induced by TAC treatment. A‐B, The mice were treated as in Figure 2A, and the NRCMs were treated as in Figure 1C. Immunoprecipitation was performed using anti‐PTEN cross‐linked magnetic beads. Precipitated proteins were immunoblotted with anti‐PTEN and anti‐Ub antibodies. C, NRCMs were transfected with ad‐TRIM10 and then stimulated by PE for 24 h with or without preteasome inhibitor MG132 (10 μmol/L). Data are mean ± SEM (n = 3‐4).*P < .05 vs PE; ^#^ *P* < .05 vs PE+MG132. D, Graphic representation of TRIM10 regulates cardiac hypertrophy via targeting PTEN/AKT pathway.TRIM10 expression is up‐regulated in cardiomyocytes by pro‐hypertrophic stimulus, such as stress and PE. TRIM10 may directly bind to PTEN and promotes PTEN ubiquitination and degradation, leading to the activation of AKT. Collectively, these findings imply that TRIM10 represents a potential therapeutic target for the prevention of pathological cardiac hypertrophy

To determine whether TRIM10 could affect PETN stability by their mutual interaction, Co‐IP assays were performed. As shown in Figure 5A,B, Co‐IP results revealed that either TAC or PE stimulation increased PTEN ubiquitination, but TRIM10 knockout or knockdown reversed this effect. In addition, the effect of MG132 (a specific proteasome inhibitor) was used to investigate TRIM10‐induced reductions in PTEN. The results showed that TRIM10 overexpression decreased PTEN expression, and MG132 reversed the reduction in PTEN (Figure 5C). Collectively, these results indicate that TRIM10 may lead to ubiquitination and degradation of PTEN, which could be an underlying reason for AKT signalling inhibition in cardiac hypertrophy processes (Figure 5D).

## 4 DISCUSSION

In the present study, we identified a novel role of TRIM10 in cardiac hypertrophy. Knockout of TRIM10 in mice inhibited hypertrophic remodelling after TAC surgery, and this effect was further verified in cardiomyocytes in vitro. In contrast, overexpression of TRIM10 in cardiomyocytes aggravated this effect in vitro. Mechanistically, we identified PTEN, a negative modifier of the PI3K/AKT/mTOR pathway, as a TRIM10 target protein. TRIM10 may directly bind to PTEN and promote its ubiquitination, consequently resulting in its proteasomal degradation and activation of hypertrophic signalling, as illustrated in Figure 5D.

TRIM family proteins (many of which have E3 ubiquitin ligase activity) have been demonstrated as key components involved in cardiomyocyte differentiation and apoptosis and play an important role in dilated cardiomyopathy and cardiac hypertrophy/atrophy/ischemia.^8^, ^20^ TRIM63 (MuRF1) overexpression induced thinning of the left ventricular wall and deterioration of cardiac function, ultimately leading to heart failure.^21^ TRIM24 promoted Rho‐dependent serum‐response factor activation and hypertrophy resulting from dysbindin in NRCMs.^22^ TRIM8 contributed to pathological cardiac hypertrophy by prompting the activation of transforming growth factor β‐activated kinase 1‐dependent signalling.^9^ TRIM32 inhibited TAC‐induced pathological cardiac hypertrophic remodelling by inhibiting Akt‐dependent signalling.^23^ Although it is reportedly involved in terminal erythroid cell differentiation and survival, at present, there are few studies on TRIM10. Recently, Huang et al found that silencing of TRIM10 reduced cell apoptosis and ROS levels in a cellular model of Parkinson's disease.^24^ However, little is known about functional roles of TRIM10 in regulating cardiac hypertrophy. At a biochemical level, it is generally accepted that similar structures perform similar functions; thus, TRIM10 may be involved in cardiac hypertrophy. In this study, our results demonstrated that hypertrophic stimuli increased TRIM10 expression in cardiomyocytes and hearts (Figure 1A,B). Knockout of TRIM10 in mice prevented cardiac hypertrophy induced by hypertrophic stimulation, and this effect was further verified in NRCMs. However, TRIM10 overexpression in cardiomyocytes aggravated this effect (Figures 1 and 2). These results strongly suggest the involvement of TRIM10 in pathological cardiac hypertrophy.

Numerous intracellular signalling pathways participate in cardiac hypertrophy accompanied by increased protein synthesis, such as IGF1R/PI3K/AKT, EGFR/ MAPKs, gp130/Jak/STAT3 and calcineurin/NFAT.^25^ Thus, we examined changes in these signalling pathways. Our results show that TRIM10 knockout had no influence on the expression of MAPKs or calcineurin; however, it significantly decreased the phosphorylation of AKT and STAT‐3 induced by TAC. As dual protein/lipid, PTEN dephosphorylates PIP3 to inhibit AKT action,^18^ and STAT3 activation can be negatively regulated by Src homology region 2 (SH‐2) domain‐containing phosphatase 1(SHP1)^19^; we next examined expression of PTEN and SHP1. The results showed that TRIM10 knockout or knockdown significantly attenuated PTEN expression, but had no effect on SHP1 expression (Figure 4A,B). Furthermore, we measured the interaction between TRIM10 and PTEN and found that TRIM10 knockout or knockdown decreased TAC‐induced PTEN ubiquitination and protein level. In addition, MG132 (proteasome inhibitor) reversed the reduction in PTEN induced by TRIM10 overexpression. These results indicate that TRIM10 may directly act on PTEN, leading to its enhanced degradation.

In summary, the present study discovered a novel role for TRIM10 in hypertrophic cardiac remodelling. Knockout or knockdown of TRIM10 accounts, at least in part, for hypertrophic remodelling of cardiac tissue induced by TAC or PE stimulation; however, overexpression of TRIM10 aggravated this response in NRCMs in vitro. Furthermore, our results verified that TRIM10 may directly bind to PTEN and promote its ubiquitination and degradation, leading to activation of AKT. Thus, our findings suggest that TRIM10 is a prospective therapeutic target for the prevention of pathological cardiac hypertrophy.

## CONFLICT OF INTEREST

The authors declare that there are no conflicts of interest.

## AUTHOR CONTRIBUTIONS

Hui Yang and Xiao‐Xiao Wang contributed to the collection of data, data analysis and interpretation, and article writing; Chun‐Yu Zhou, Xue Xiao, Cui Tian and Chun‐Lin Yin contributed to the collection of data, as well as data analysis and interpretation; Hui‐Hua Li and Hong‐Xia Wang provided financial support and contributed to conception and design, and manuscript writing; Hong‐Xia Wang provided administrative support and final approval of the manuscript.

## Supporting information

Table S1

## Data Availability

The data that support the findings of this study are available on request from the corresponding author.
