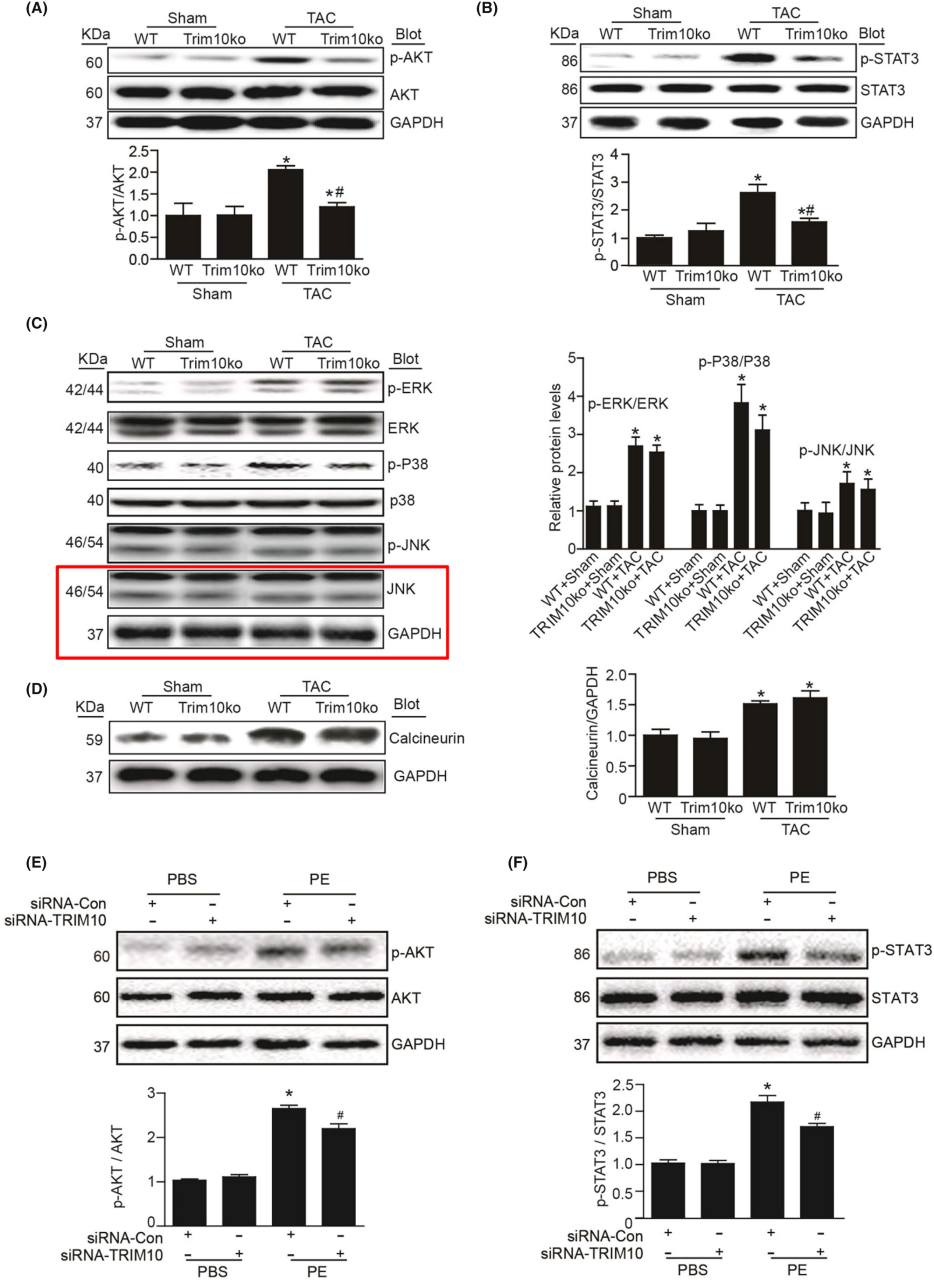#  

**DOI:** 10.1111/jcmm.17773

**Published:** 2023-07-02

In the article,^1^ the published article contains errors in Figures 2E and 3C.

In Figure 2E, bottom left panels, the image in WT+Sham was the same as TRIM10ko+Sham. In Figure 3C, last 2 panels, the image of p‐JNK was the same as the image of JNK.

The correct figures are shown below. The authors confirm all results and conclusions of this article remain unchanged.

FIGURE 2. TRIM10 deficiency alleviates TAC‐induced cardiac hypertrophy. A, TRIM10ko and WT mice were subject to TAC or Sham surgery for 2w. Ejection fraction (EF%) and fractional shortening (FS%) were assessed by M‐mode echocardiograms. B, The representative heart photographs of each group were showed, and heart weight/body weight (HW/BW) and heart weight/tibia length (HW/TL) ratios were calculated. C, The cell size was examined by WGA staining. D, The mRNA levels of hypertrophic markers such as ANF, BNP and β‐MHC were detected by qPCR. E, The interstitial fibrosis areas were calculated by Masson's staining of heart sections (×10 and ×200). F, The mRNA levels of collagen I and III in the hearts were detected by qPCR. G, The apoptosis were green; myocardial tissue were identified by a‐actinin antibody staining (red) and nuclei by DAPI staining (blue) (×200). Data are mean ± SEM (*n* = 6 per group). *p < 0.05 vs. Sham group, #*p* < 0.05 vs. TAC group.
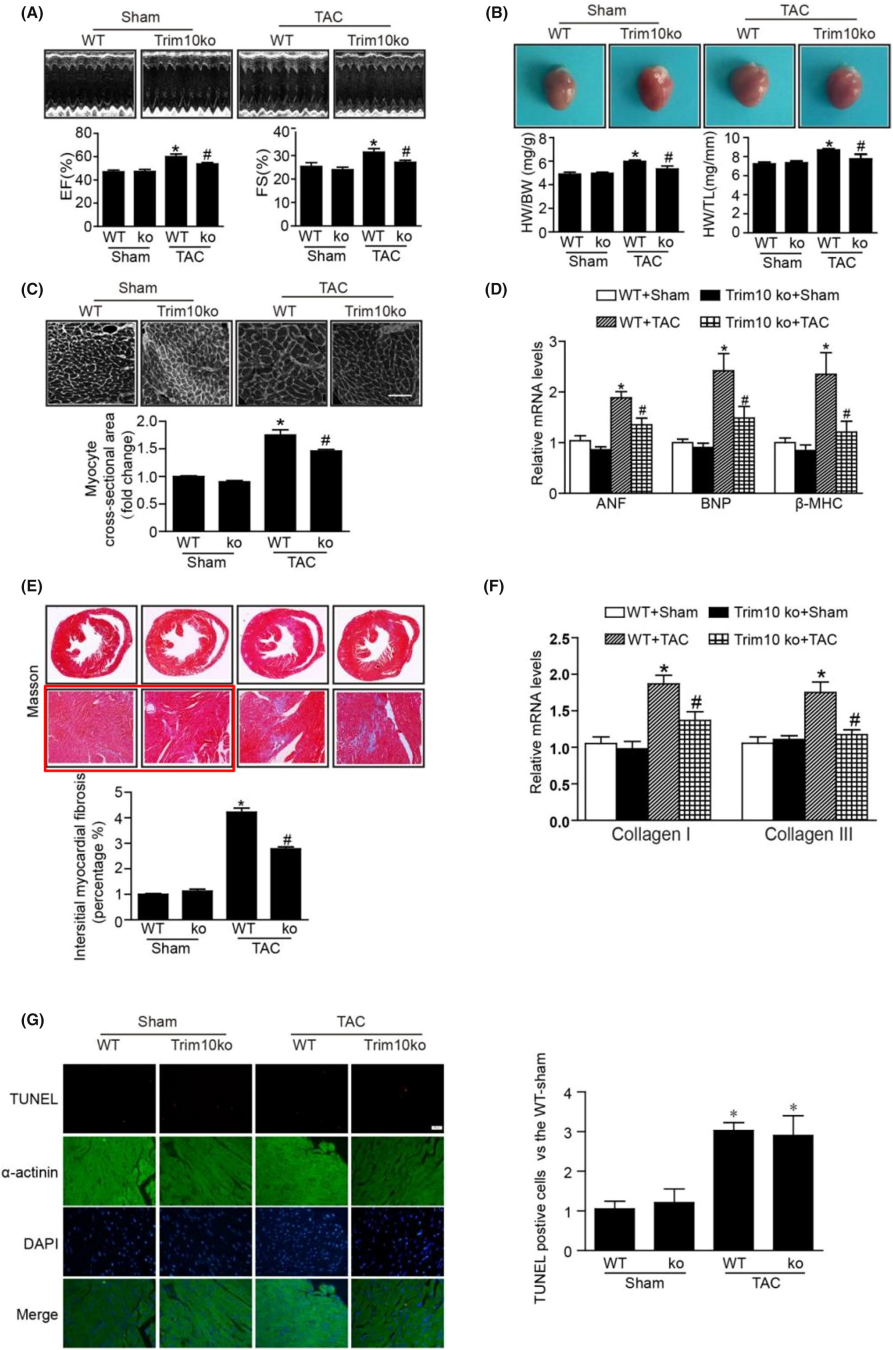


FIGURE 3. TRIM10 deficiency or knockdown inhibits the activation of AKT and STAT3 signalling pathways. A, The ratio of p‐AKT/AKT was analysed in the hearts of mice treated as in Figure 2A. B, The ratio of p‐STAT/STAT. C, The ratios of p‐ERK1/2/ERK1/2, p‐P38/P‐38 and p‐JNK/JNK. D, The expression of calcineurin. E, NRCMs were treated as in Figure 1C. The ratio of p‐AKT/AKT in NRCMs treated as in Figure 1C. F, The ratio of p‐STAT/STAT in NRCMs treated as in Figure 1C. GAPDH was used as a loading control. Data are mean ± SEM (*n* = 3–4). *p < 0.05 vs. Sham or PBS group; #*p* < 0 0.05 vs. TAC group or PE group.