# Supplementary material for: Tripartite motif 10 regulates cardiac hypertrophy by targeting the PTEN/AKT pathway
Source: J Cell Mol Med. 2020 Apr 28;24(11):6233–41. doi: 10.1111/jcmm.15257 (PMC7294125; doi:10.1111/jcmm.15257)
Supplement: Supplementary file 1 — Table S1 [file JCMM-24-6233-s001.docx]

**Table S1**. Primer sequences for RT-PCR analysis

| Gene symbol | Forward primer (5’-3’) | Reverse primer (5’-3’) |
| --- | --- | --- |
| ANF (rat) | CTTCTCCATCACCAAGGGCTT | GGATTTGCTCCA ATATGGCCT |
| BNP (rat) | TGATTCTGCTCCTGCTTTTC | GTGGATTGTTCTGGAGACTG |
| β-MHC(rat) | CGAGGCAAGCTCACGTATAC | CTTGGCTTCTGTTTCCTCCT |
| ANF (mouse) | CACAGATCTGATGGATTTCAAGA | CCTCATCTTCTACCGGCATC |
| BNP (mouse) | GAAGGTGCTGTCCCAGATGA | CCAGCAGCTGCA TCTTGAAT |
| β-MHC(mouse) | GATGTTTTTGTGCCCGATGA | CAGTCACCGTCTTGCCATTCT |
| Collagen I (mouse) | GAGTACTGGATCGACCCTAACC | GACGGCTGAGTAGGGAACACAA |
| Collagen III (mouse) | TCCCCTGGAATCTGTGAATC | TGAGTCGAATTGGGGAGAAT |
| GAPDH (rat) | GGC AAGTTC AAT GGC ACAGT | TGGTGA AGACGCCAGTAG ACTC |
| GAPDH(mouse) | GGTTGTCTCCTGCGACTTCA | GGTGGTCCA GGGTTTCTTACTC |
